# Worldwide Tinnitus Research: A Bibliometric Analysis of the Published Literature Between 2001 and 2020

**DOI:** 10.3389/fneur.2022.828299

**Published:** 2022-01-31

**Authors:** Fangwei Zhou, Tian Zhang, Ying Jin, Yifei Ma, Zhipeng Xian, Mengting Zeng, Guodong Yu

**Affiliations:** ^1^Department of Clinical Medicine, Guizhou Medical University, Guiyang, China; ^2^Department of Otorhinolaryngology Head and Neck Surgery, Affiliated Hospital of Guizhou Medical University, Guiyang, China

**Keywords:** tinnitus, CiteSpace, VOSviewer, bibliometric analysis, hotspot, trend

## Abstract

**Background:**

In recent years, tinnitus has attracted increasing research interest. However, bibliometric analysis of global research on tinnitus is rare. The objective of this study was to identify and describe the foci and developing trends of tinnitus research using a bibliometric approach.

**Methods:**

Publications related to tinnitus published from 2001 to 2020 were searched for in the Science Citation Index-Expanded (SCI-E) and Social Sciences Citation Index (SSCI) databases in the Web of Science Core Collection of Clarivate Analytics. The bibliometric approach was used to estimate the searched data, and VOSviewer and CiteSpace software were used to identify and analyze research foci and trends in the field of tinnitus.

**Results:**

A total of 5,748 articles were included. The number of publications on tinnitus has increased dramatically in the last 20 years, especially since 2010. The leading country in terms of publications and access to collaborative networks was the United States. High-frequency keywords included tinnitus, hearing loss, prevalence, management, depression, mechanism, vertigo, hearing, inferior colliculus, and noise. The analyses of keyword burst detection indicated that prevalence, anxiety, and neural network are emerging research hotspots.

**Conclusion:**

In the past 20 years, academic understanding of tinnitus has improved considerably. This study provides an objective, systematic, and comprehensive analysis of tinnitus-related literature. Furthermore, current hot spots and prospective trends in the field of tinnitus were identified. These results will assist otolaryngologists and audiologists in identifying the evolving dynamics of tinnitus research and highlight areas for prospective research.

## Introduction

Tinnitus is a prevalent chronic health condition defined as a perception of sound in the ear and/or head in the absence of external stimuli (1). These auditory perceptions are often described as whistles, hisses, or buzzing sounds. Tinnitus may be associated with acoustic trauma (such as exposure to loud noises), chronic hearing loss, emotional stress, or spontaneous occurrence (2). Epidemiological studies have shown that the prevalence of tinnitus in adults is 10–15% (3). Approximately 3–5% of the general adult population finds tinnitus sufficiently troublesome to interfere with sleep and mood, making it difficult to perform daily activities (4). Although the overall economic impact of tinnitus remains unclear, untreated severe tinnitus may be correlated with a significant economic cost to society. A previous study suggested that the average annual cost of treatment for tinnitus in the United Kingdom (UK) is approximately £750 million (5). Some patients suffer from severe tinnitus, which can be associated with frustration, irritability, anxiety, depression, cognitive dysfunction, insomnia, stress, and emotional exhaustion—all of which contribute to a significant decline in quality of life (6).

Tinnitus is often considered to be a chronic disorder that is difficult to treat. Various treatment protocols have been proposed to alleviate tinnitus, including sound masking, hearing aids, medication, counseling, and acupuncture (7, 8). Although significant progress has been made in this area of research, current treatments have failed to cure the disease. Additionally, the majority of treatments have limited effectiveness in treating tinnitus-related disability and distress. Therefore, more efficient therapies with the least side effects are urgently needed to reduce the suffering caused by tinnitus. During the past two decades, scholars around the world have made enormous advances in the study of the epidemiology, diagnosis, pathophysiology, and treatment of tinnitus. However, there is an absence of visual overviews that can assist researchers in obtaining research trends in the field of tinnitus.

Bibliometric analysis is a novel method for quantitatively assessing the impact of research literature on selected research areas over specific time periods, countries/regions, research collaborations, journals, institutions, and authors (9, 10). Bibliometric analysis has been widely applied to a series of research domains (11). By comparing changes in research hotspots over time, it presents an accessible way of revealing research hotspots and predicting the orientation of the chosen research area in the future. In contrast to traditional systematic reviews, bibliometric analysis provides a current, intuitive, and unbiased way to track developments and explore particular areas of knowledge (12). CiteSpace is a Java-based scientific mapping software for bibliometrics and comparative analysis. VOSviewer is a novel utility for building maps based on web data, and then visualizing and exploring these maps (13). Recently, VOSviewer and CiteSpace have been employed in a variety of fields such as healthcare, sports management, and urban development (14, 15).

Bibliometric techniques have not yet been used to summarize the literature on tinnitus. Moreover, relatively little research has predicted tinnitus hotspots. In this study, we intended to visually analyze tinnitus research hotspots through the bibliometric approach from databases over the past 20 years. We briefly reviewed the current tinnitus research hotspots and predicted potential development trends in this field in the next few years. These analyses offer otolaryngologists and audiologists a comprehensive insight into the macroscopic and microscopic characteristics of the entire knowledge domain.

## Materials and Methods

### Data Source and Search Strategy

**Figure 1:**
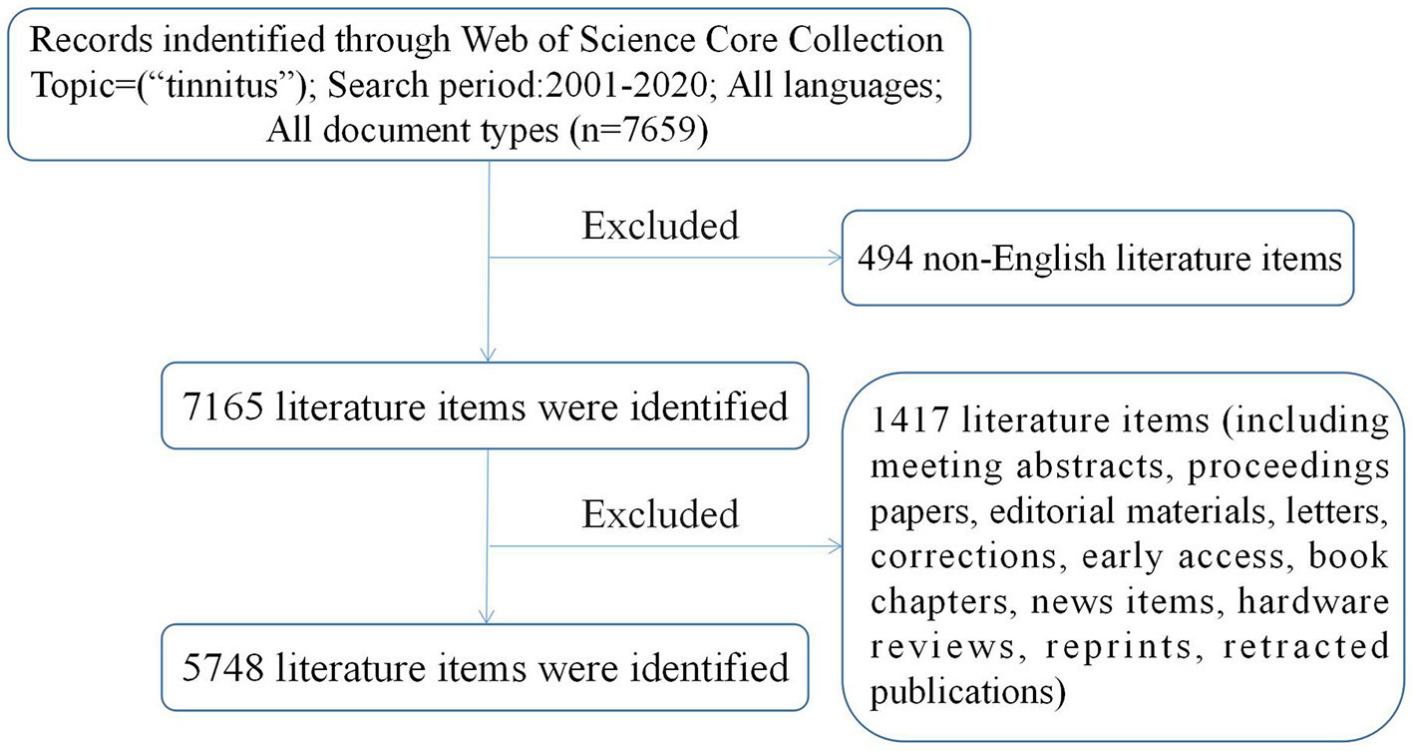
Frame flow diagram of search strategy.

The Science Citation Index-Expanded (SCI-E) and Social Sciences Citation Index (SSCI) of the Web of Science Core Collection (WoSCC) were utilized for the bibliometric analysis. We thoroughly searched the WoSCC database for relevant data published between 2001 and 2020, and included original articles only. The retrieval strategy can be stated as: Topic = (tinnitus) AND Language = English. To preclude potential bias from frequently updated databases, all data downloads and literature searches were completed on October 1, 2021. The detailed search procedure is shown in Figure 1. Two researchers undertook the data analysis independently. Any divergences were settled by consultation or by seeking the help of external specialists to reach consensus. We documented information on titles, abstracts, key words, authors, institutions, countries, journals, references, and citations.

### Analysis Method

All WoSCC data were transformed into text and then imported into the analysis software. VOSviewer 1.6.16 (Leiden University, Leiden, Netherlands), CiteSpace 5.8. R3, 64-bit (Drexel University, Philadelphia, USA), and the bibliometric online analysis platform (http://bibliometric.com/) were applied to identify co-cited articles, keywords, countries, institutions, journals, authors, and network characteristics of keyword bursts, and to present the results visually. The H-index, impact factor, and category quartiles were all obtained from the Journal Citation Report 2020. We interrogated the H-index, which is considered an essential metric for assessing the scientific impact of journals, authors, or countries (16, 17).

To survey the hotspots of tinnitus research, we analyzed the identified literature in series using CiteSpace software, including publication institutions, co-cited references, and the most correlated keywords. On the constructed network visual map, the nodes represented the objects analyzed, and objects with high frequency were represented by larger nodes. Furthermore, we performed an analysis using the CiteSpace software for centrality, which is an index that defines the significance of the network nodes, where more prominent nodes are represented by higher centrality (18). Centrality is utilized to quantify the importance of a node's position in the network. The higher the centrality, the greater the number of connections in the network that pass through that node.

VOSviewer can be used to construct scientifically based knowledge networks that display the evolution of research domains, show inter-institutional collaborations, and foreshadow upcoming research hotspots. In this study, we analyzed the co-occurrence of keywords visually and constructed density maps using VOSviewer. Co-occurrence analysis in VOSviewer can categorize keywords into distinct clusters, where different clusters are marked with different colors. Cluster analysis of research hotspots can be optimized to visualize and identify the trend of development through a keyword co-occurrence network.

## Results

### Article Distribution by Publication Year

**Figure 2:**
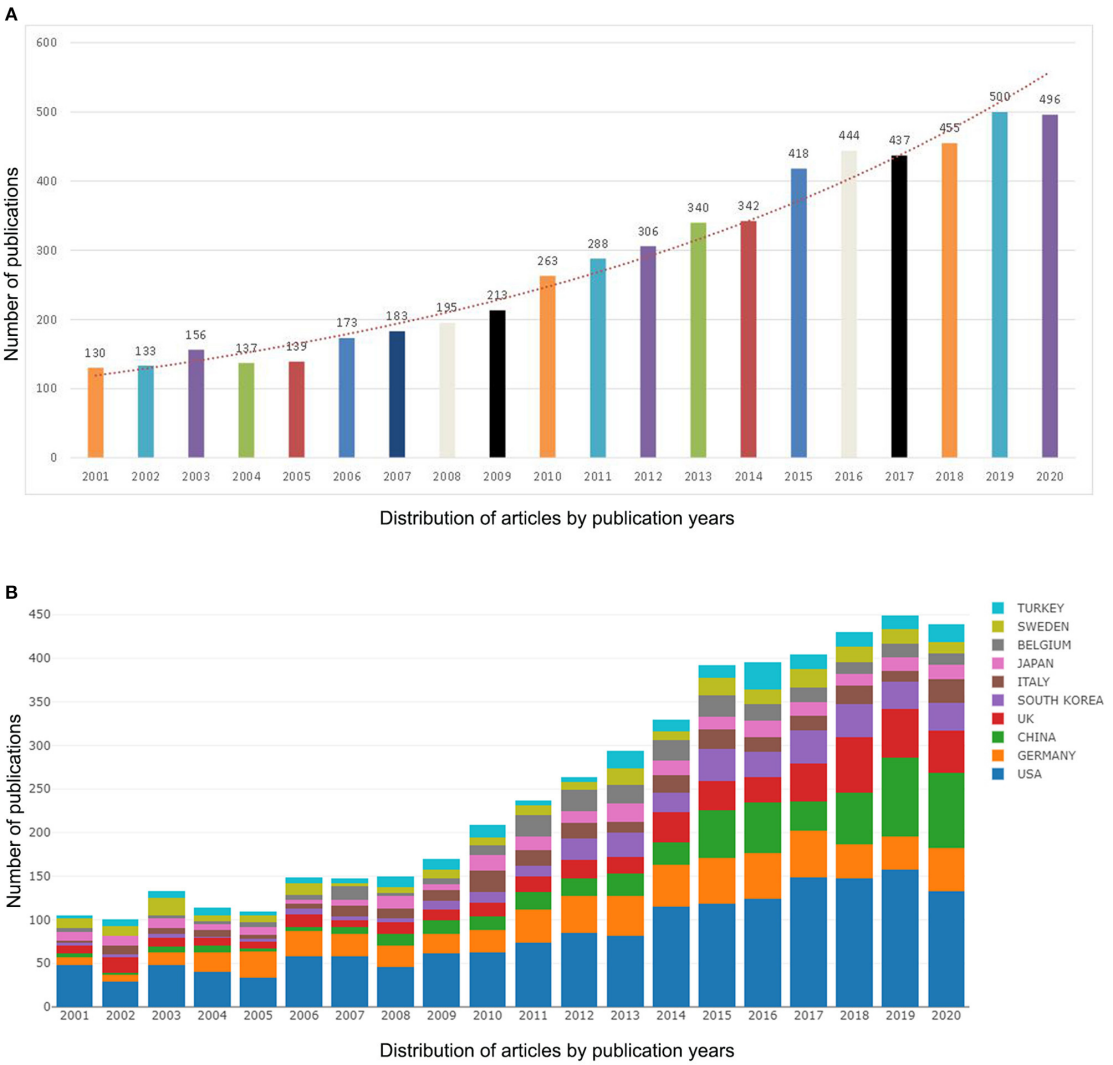
Trends in the number of publications **(A)** and the top 10 countries/regions **(B)** on tinnitus research from 2001 to 2020.

A total of 5,748 original articles were published between 2001 and 2020. Tinnitus-related research output exhibited an overall upward trend between 2001 and 2020 (Figure 2A). The number of articles published by United States (US) academics was the highest in the last 20 years (Figure 2B). From 2015 onwards, the number of papers on tinnitus increased sharply, with more than three times the number of papers in 2020 than in 2001. From 2015 to 2020, research activity on tinnitus peaked in terms of volume, with 2750 papers being published in 6 years, almost half of the total.

### Distribution of Countries/Regions and Institutions

**Figure 3:**
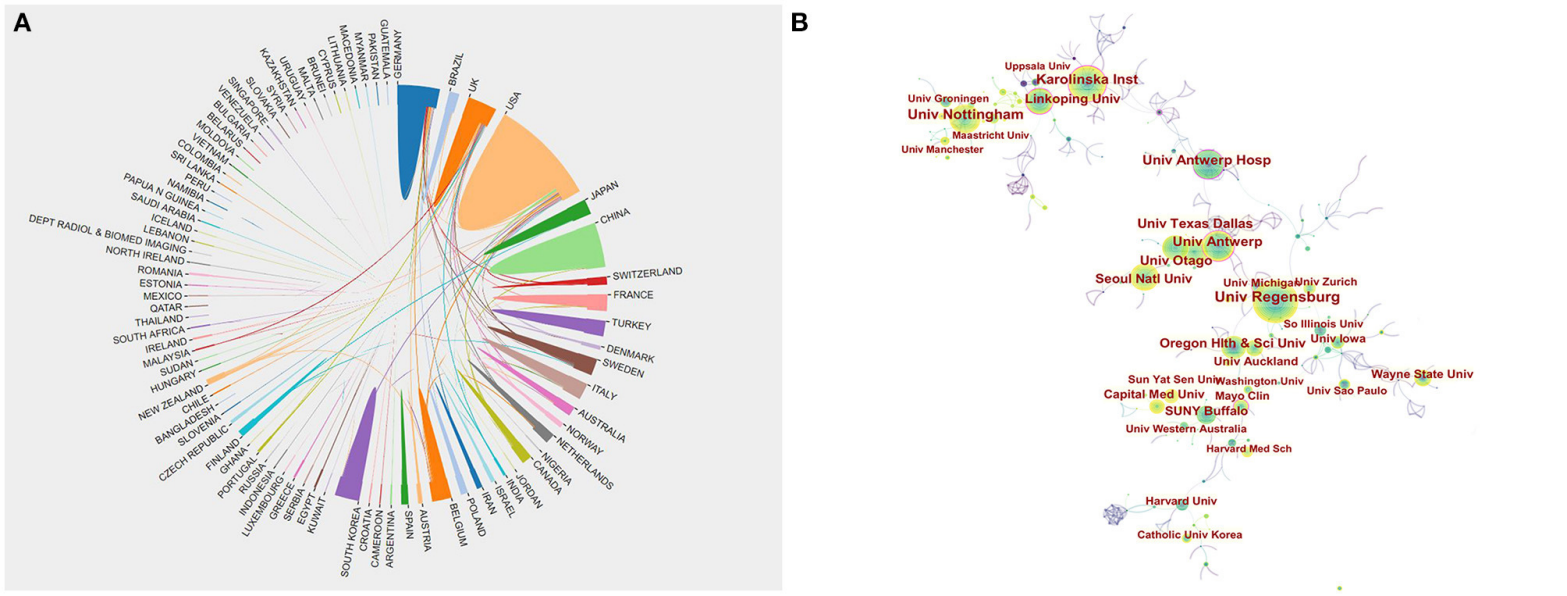
The co-operation of countries/regions **(A)** and institutions **(B)** contributed to publications on tinnitus research from 2001 to 2020. The color and thickness in the inner circle of the node indicated the occurrence frequency of different time periods.

Figure 3A illustrates the network of national collaborations for tinnitus research. Table 1 displays the top 10 contributing nations. The top contributor was the US (1688), followed by Germany (672), the UK (467), China (466), and South Korea (348). Among the top 10 countries, the US was the leading contributor to tinnitus research, publishing nearly 30% of all studies. The centrality index weighs the prominence of network nodes. Centrality analysis revealed that the US (0.23) was at the core of the network, followed by Sweden (0.18), and the UK (0.14). In a collaborative network, higher centrality equated to more intensive co-operation. The graph of country-based research networks demonstrates a lower density, suggesting relatively independent research teams and emphasizing the necessity for further collaboration.

**Table 1:** Ranking of top 10 most published countries in tinnitus research from 2001 to 2020.

**Rank**	**Country**	**Articles counts**	**Centrality**
1	USA	1,688	0.23
2	Germany	672	0.00
3	UK	467	0.14
4	China	466	0.05
5	South Korea	348	0.05
6	Italy	280	0.11
7	Japan	266	0.00
8	Belgium	257	0.00
9	Turkey	256	0.09
10	Sweden	254	0.18

Figure 3B presents the institutional co-operation network, identifying the top 10 collaborating institutions, including University of Antwerp (169), University of Regensburg (152), Karolinska Institute (141), University of Texas System (140), and University of California system (138) (Table 2). Karolinska Institute (0.08), University of Regensburg (0.07), and Harvard University (0.04), had the top centrality rankings. All institutions had low centrality, which indicated less inter-institutional collaboration.

**Table 2:** Ranking of top 10 institutions for collaboration in tinnitus research from 2001 to 2020.

**Rank**	**Institutions**	**Articles counts**	**Country**	**Centrality**
1	University of Antwerp	169	Belgium	0.03
2	University of Regensburg	152	German	0.07
3	Karolinska Institutet	141	Sweden	0.08
4	University of Texas System	140	USA	0.00
5	University of California system	138	USA	0.00
6	University of Nottingham	135	UK	0.04
7	Harvard University	114	USA	0.04
8	U.S. Department of Veterans Affairs	105	USA	0.01
9	Veterans Health Administration	105	USA	0.02
10	State University of New York	101	USA	0.00

### Contributions of Authors

**Figure 4:**
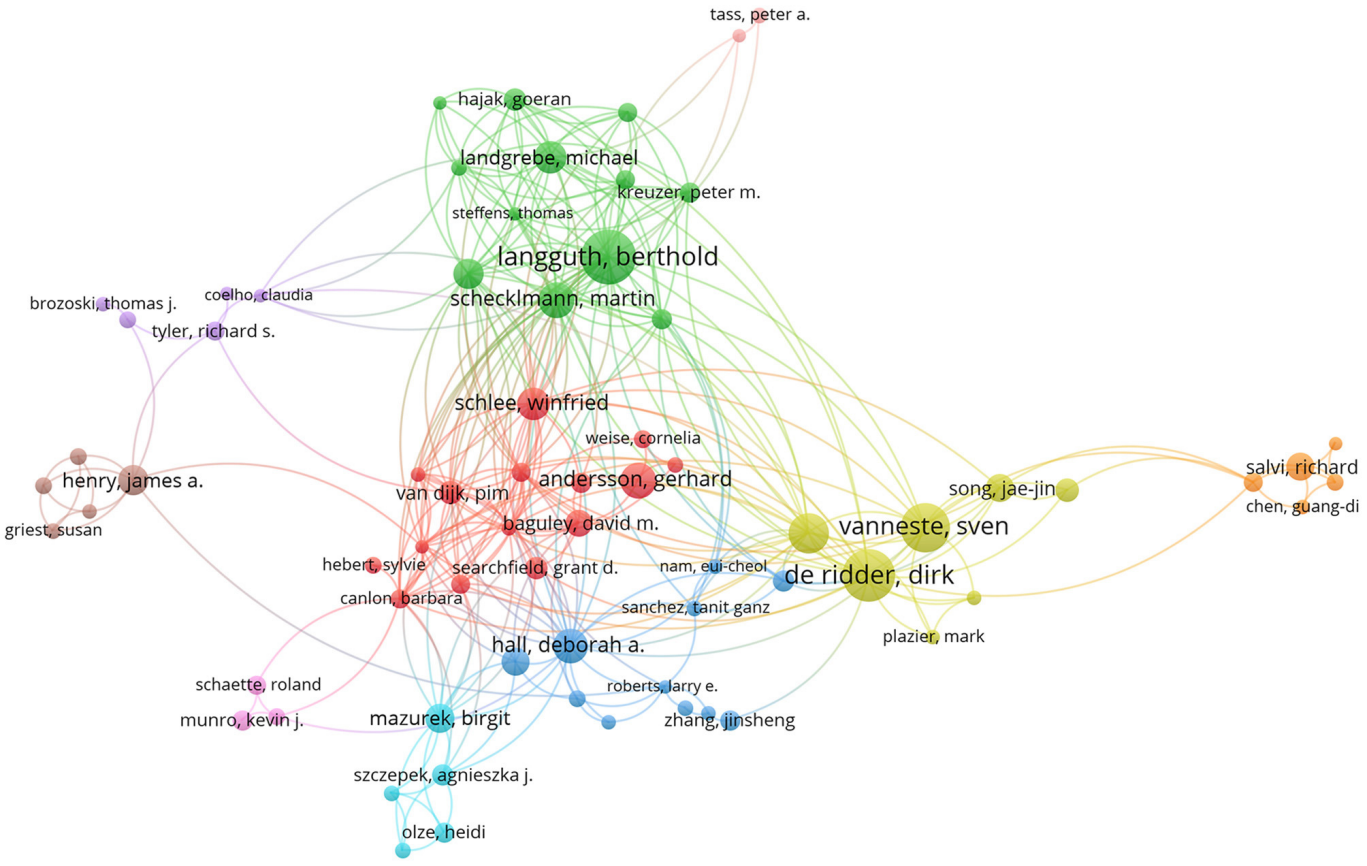
Joint mapping of productive authors in tinnitus research from 2001 to 2020. The size of each colored circle is proportional to the total number of articles published by the author.

The use of a visual map of co-authored publications allows for the identification of research groups and potential collaborators with the greatest impact, which can also help to establish collaborative relationships among researchers. A visualization of authors with at least 10 publications and a total of at least 300 citations was performed using VOSviewer, as shown in Figure 4. The map consists of 82 circles, each representing an author. Some names may not be visible due to the overlap of the names. Closed circles signify active authors with strong study collaborations.

Based on our dataset, 19 893 authors published papers on tinnitus from 2001 to 2020. The top 10 most prolific authors during the study period are listed in Table 3. The author with the greatest number of publications was D De Ridder from University Hospital Leuven (134 articles; 7,197 citations), while B Langguth from the University of Regensburg narrowly occupied second place (132 articles; 6,519 citations). In terms of centrality, M Schecklmann of the University of Regensburg occupied first place (0.04). The centerlines of all study groups are less than 0.1, suggesting that the research teams are weakly collaborative in tinnitus research and need to further strengthen their cooperation.

**Table 3:** Ranking of top 10 most published authors in tinnitus research from 2001 to 2020.

**Rank**	**Author**	**Articles counts**	**Centrality**	**Total citations**	**Average citations**	**H-index**
1	De Ridder D	134	0.00	7,073	52.78	43
2	Langguth B	132	0.00	6,417	48.61	39
3	Vanneste S	108	0.00	3,885	35.97	35
4	Van De Heyning P	96	0.01	3,159	32.91	33
5	Andersson G	72	0.00	3,004	41.72	28
6	Hall DA	59	0.00	1,361	23.07	23
7	Schecklmann M	58	0.04	1,837	31.67	22
8	Kleinjung T	50	0.00	2,288	45.76	26
9	Landgrebe M	50	0.03	1,936	38.72	25
10	Schlee W	50	0.04	1,759	35.18	20

### Journal Analysis

Table 4 illustrates the features of the 10 most active journals. Most of the publishers of these journals are located in the US. The highest number of articles related to tinnitus were published by *Otology & Neurotology, Hearing Research*, and *International Journal of Audiology*. The journal *Laryngoscope* has also released a plentiful quantity of articles on tinnitus with a high impact factor. In addition, *Hearing Research* had the highest average number of citations (32.46) and the highest H-index (51). The Journal Citation Report quartile Q1 contained *Ear and Hearing*; Q2 included *European Archives of Oto-Rhino-Laryngology, PLoS One, Laryngoscope*, and *Otolaryngology-Head and Neck Surgery*; and *Otology & Neurotology, Hearing Research*, and *International Journal of Audiology* were listed as Q3.

**Table 4:** Ranking of top 10 journals for number of published articles on tinnitus research from 2001 to 2020.

**Journal**	**Articles counts**	**Country**	**Journal citation reports (2020)**	**Impact factor (2020)**	**Total cites**	**Average number of citations**	**H-index**
Otology & Neurotology	334	USA	Q3	2.311	6,973	20.88	42
Hearing Research	226	Netherlands	Q3	3.208	7,336	32.46	51
International Journal of Audiology	189	UK	Q3	2.117	3,717	19.67	34
Acta Oto-Laryngologica	155	UK	Q4	1.494	2,725	17.58	27
European Archives of oto-rhino-laryngology	152	German	Q2	2.503	2,264	14.89	25
Plos One	142	USA	Q2	3.240	3,510	24.72	32
Laryngoscope	140	USA	Q2	3.325	3,056	21.83	31
Journal of Laryngology And Otology	118	UK	Q4	1.469	1,135	9.62	17
Otolaryngology-Head And Neck Surgery	96	USA	Q2	3.497	2,980	31.04	29
Ear And Hearing	87	USA	Q1	3.570	2,404	27.63	30

### Cluster Analysis of Keyword Co-occurrence Related to Research Hot Spots

**Figure 5:**
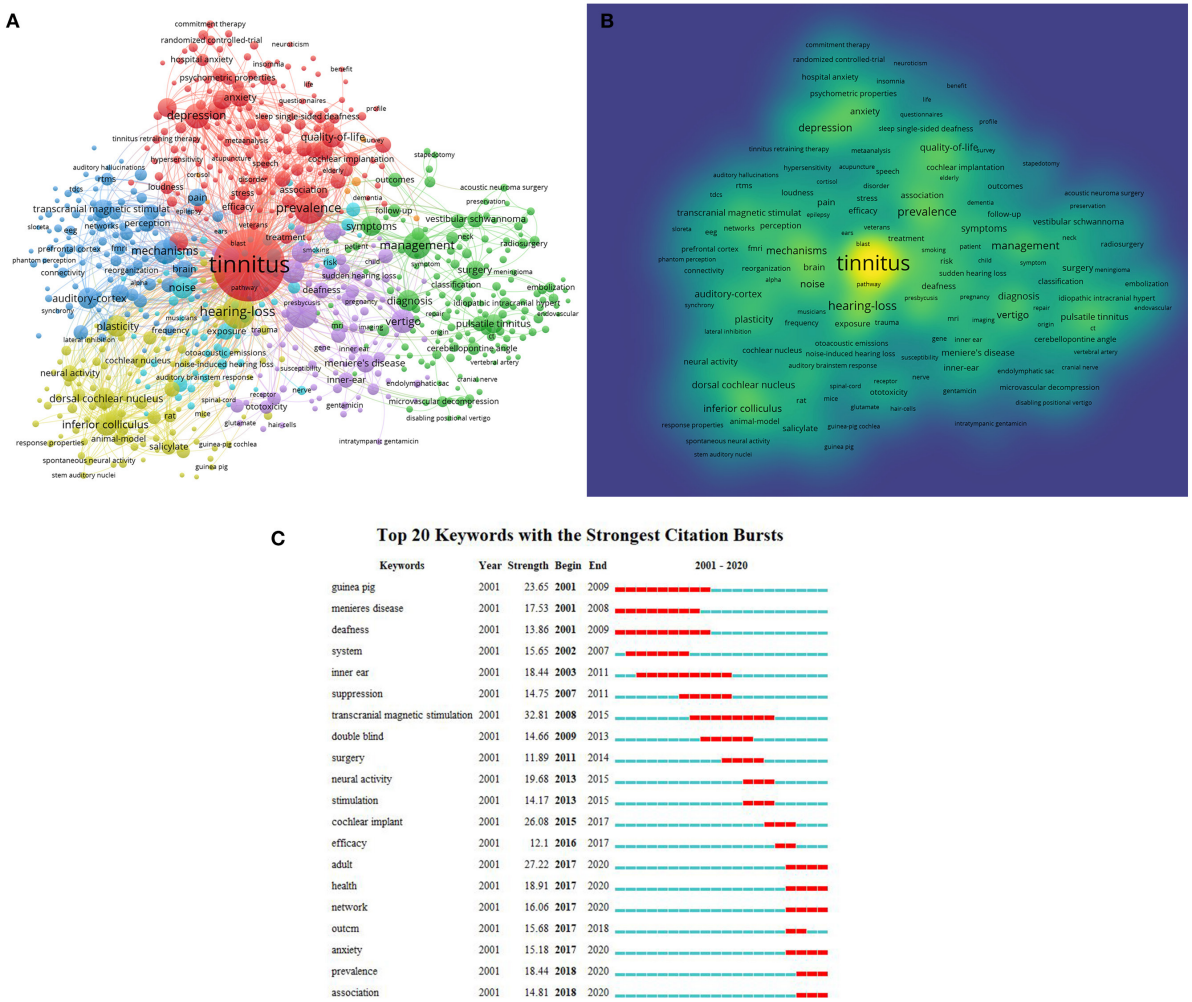
Co-occurrence analysis of global research on tinnitus based on the WoSCC database from 2001 to 2020. **(A)** Mapping of keywords in the research field. **(B)** Distribution of keywords according to average frequency of appearance. Yellow keywords represent the highest frequency, followed by green and purple. **(C)** Keywords with the strongest citation bursts in tinnitus research.

For the 5,748 articles retrieved, VOSviewer analysis was used to search the titles and abstracts for keywords. A map was then created with 408 terms (13 920 in total), with at least 20 appearances per term, which were categorized into five clusters (Figure 5A). In the map, the high-frequency keywords were tinnitus (2526), hearing loss (999), prevalence (452), management (365), depression (312), mechanisms (298), vertigo (271), hearing (258), inferior colliculus (248), and noise (236). Terms with comparable study populations were merged under the same catalog, with five major clusters of clinical characteristics, mechanisms, diagnosis, management, and pathophysiology of tinnitus. At the same time, the frequency of the keywords can be used by VOSviewer to ascertain their density, which can be presented in a density graph (Figure 5B). The warmer the color (toward yellow), the higher the density. The research hotspots in the field tend to be found in regions with larger grayscale levels.

### Burst Detection Using Keywords

Based on the analysis of the 5,748 articles identified in the WoSCC database, keyword outbreaks from 2001 to 2020 were ascertained (Figure 5C). On Figure 5C a blue line cutting the year depicts the timeline, and the burst period is illustrated by a red reflective line, which indicates the beginning and ending years, as well as the citation burst time span. Keywords with little or no research significance were excluded to focus on keywords that represented trends in tinnitus research. Between 2001 and 2020, transcranial magnetic stimulation had the highest burst strength (32.81), followed by adult (27.22), cochlear implant (26.08), guinea pig (23.65), and neural activity (19.68).

### Most Frequently Co-cited Articles and the Analysis of Their Clusters

**Figure 6:**
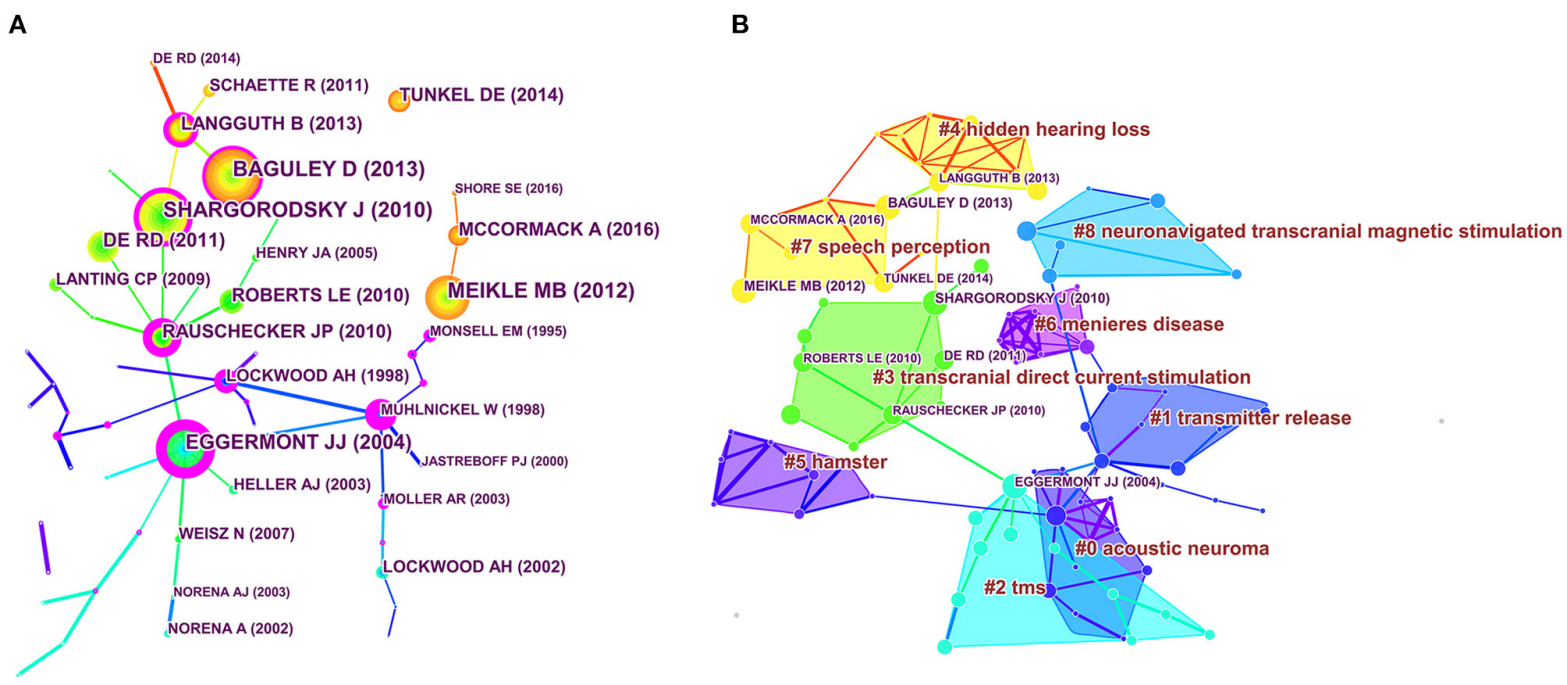
Co-cited references map **(A)** and clustered network map of co-cited references **(B)** on tinnitus research from 2001 to 2020. The diameter of each node is proportional to the total co-citation counts of the associated article. The thick purple circles in the nodes indicated high intersexual centrality.

In this study, 45 702 cited references from 5,748 articles were analyzed for co-citation relevance, from which a cluster network graph was obtained. Figure 6A displays the visual network of the co-cited articles consisting of 84 nodes and 116 links. Each node represents a cited article. The citation frequency by the same article is indicated by the links between the nodes. The node diameter is proportional to the total number of articles co-cited. The phase of development of a domain can be bridged by the nodes, where high centrality is represented by a thick purple ring. The red ring denotes the blast of citations. In the next step, study hotspots were defined by hierarchically ranking the co-cited articles produced in the co-citation network.

**Figure 7:**
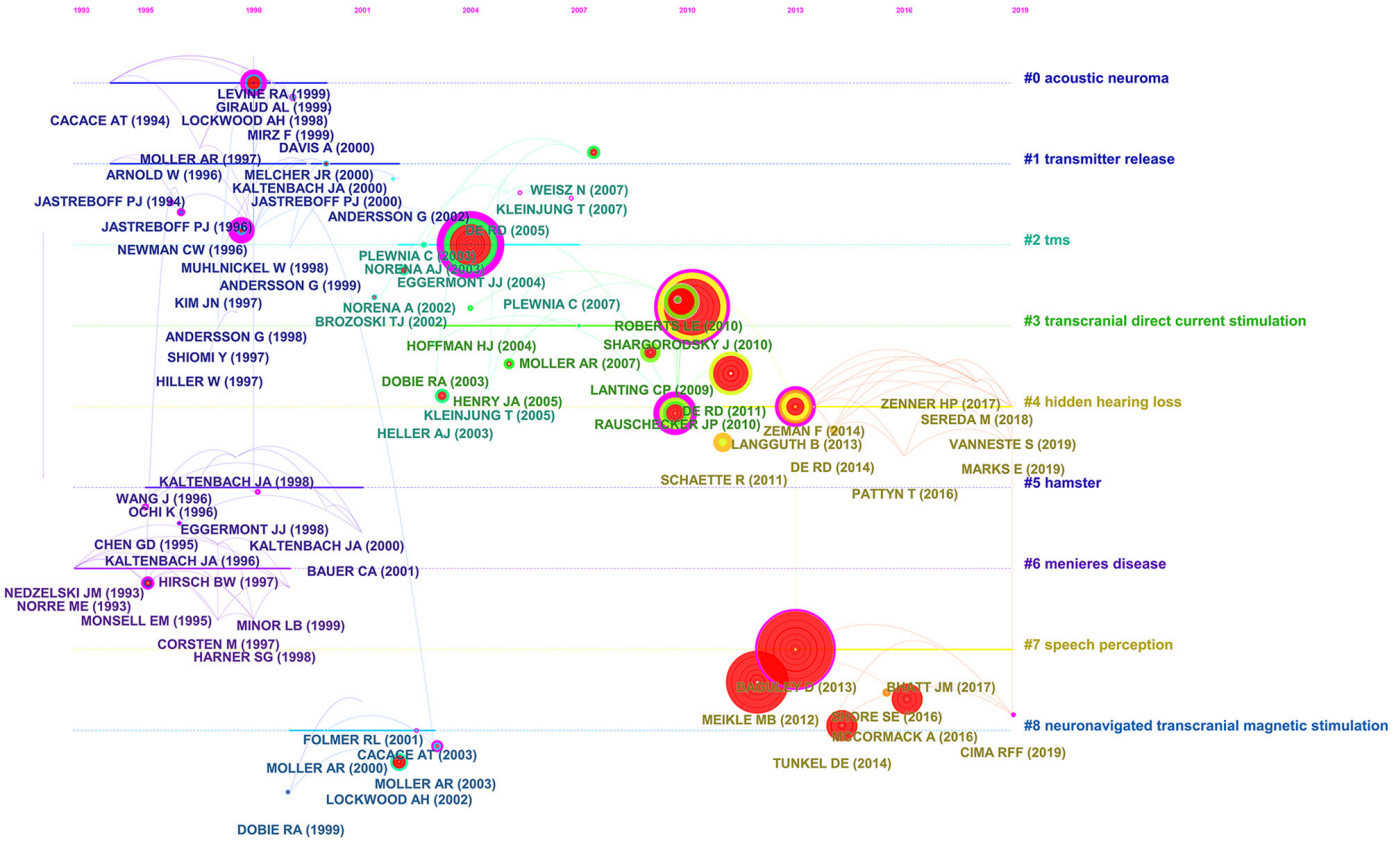
The timeline view of co-cited clusters with cluster labels. This view clearly presents the differences in the appearance time point and time span of nine clusters.

There were nine main clusters of co-cited references, consisting of acoustic neuroma, transmitter release, transcranial magnetic stimulation (TMS), transcranial direct current stimulation, hidden hearing loss, hamster, Meniere's disease, speech perception, and neuronavigated transcranial magnetic stimulation (Figure 6B). A timeline perspective of the clustering diagram is demonstrated in Figure 7, which provides support for the findings of emerging research hotspots in tinnitus. The top 10 co-cited papers are listed in Table 5. The work by Kujawa and Liberman (19) was the most quoted in *Journal of Neuroscience Methods* (1280 citations), followed by Lefaucheur et al. (20) in *Clinical Neurophysiology* (975 citations), and Poreisz et al. (21) in *Brain Research Bulletin* (669 citations).

**Table 5:** The top 10 most co-cited references on tinnitus research from 2001 to 2020.

**Rank**	**Title**	**Cited frequency**	**Author**	**Year**	**Journal**
1	Adding Insult to Injury: Cochlear Nerve Degeneration after “Temporary” Noise-Induced Hearing Loss	1280	Kujawa and Liberman (19)	2009	Journal of Neuroscience Methods
2	Evidence-based guidelines on the therapeutic use of repetitive transcranial magnetic stimulation (rTMS)	975	Lefaucheur et al. (20)	2014	Clinical Neurophysiology
3	Safety aspects of transcranial direct current stimulation concerning healthy subjects and patients	669	Poreisz et al. (21)	2007	Brain Research Bulletin
4	Evidence-based guidelines on the therapeutic use of transcranial direct current stimulation (tDCS)	566	Lefaucheur JP	2017	Clinical Neurophysiology
5	Guided Internet-based vs. face-to-face cognitive behavior therapy for psychiatric and somatic disorders: a systematic review and meta-analysis	507	Andersson G	2014	World Psychiatry
6	Prevalence and Characteristics of Tinnitus among US Adults	486	Shargorodsky J	2010	American Journal of Medicine
7	Tinnitus with a Normal Audiogram: Physiological Evidence for Hidden Hearing Loss and Computational Model	476	Schaette and McAlpine (22)	2011	Journal of Neuroscience
8	Diagnostic criteria for Meniere's disease	467	Lopez-Escamez JA	2015	Journal of Vestibular Research-Equilibrium & Orientation
9	Tinnitus	415	Baguley et al. (2)	2013	Lancet
10	Tuning Out the Noise: Limbic-Auditory Interactions in Tinnitus	372	Rauschecker JP	2010	Neuron

## Discussion

Tinnitus is a high-prevalence condition linked to hearing loss in the majority of cases. Medication is used extensively, with over four million off-label prescriptions for tinnitus relief in Europe and the US each year (23). Even though the currently available psychotherapies can provide relief when applied, a high proportion of tinnitus cases remain untreated and in despair, believing that they have to learn to co-exist with tinnitus (24). Therefore, it is worth summarizing the worldwide research developments in tinnitus in the last few years. This was the first time that quantitative and qualitative bibliometric analyses had been applied to tinnitus, covering 5,748 research articles searched from the WoSCC. We conducted a bibliometric analysis of the global article output in the tinnitus field, revealing major evolution between 2001 and 2020. The results suggested that the overall volume of annually published tinnitus-related research papers worldwide has progressively increased over the past decade, reflecting its growing importance as an area of research in otolaryngology.

The US leads the area, contributing 29.4% of all papers This has greatly promoted the advancement of tinnitus research. The US demonstrates the most intensive collaboration and the highest centrality to other countries. This trend partly reflects the mature medical research and health environments in these countries, and the critical demand for efficient tinnitus treatments. In addition, the larger population size obviously affects the number of publications. Germany, Japan, and Belgium also published many papers; however, they may have less co-operation with other countries, as evidenced by their relatively low centrality. We suggest that the countries with lower rates of co-operation should strengthen their focus on international collaboration, especially with the leading nations in tinnitus research, which will accelerate their progress in tinnitus study. In tinnitus research, more than half of the 10 top publishing organizations are located in the US. The Karolinska Institute, the University of Regensburg, and the University of Nottingham have the strongest partnerships with other institutions, which is beneficial to those institutions that rarely communicate with each other.

D De Ridder has produced the most publications in the domain of tinnitus. Furthermore, B Langguth, S Vanneste, P Van De Heyning, and G Andersson were the top five most productive authors of the past 20 years. D De Ridder (43), B Langguth (39), and S Vanneste (35) were the top three authors with a high H-index. Nevertheless, it is significant to note that global tinnitus researchers have a distinct geographical profile, with most of these academics working in Europe and the US. These authors work primarily in the otolaryngology departments of their university-affiliated hospital. Therefore, strengthening communication and co-operation among global researchers would facilitate the production of tinnitus research.

The most published journals in tinnitus are the leading journals in the field of otolaryngology and audiology, including *Otology & Neurotology, Hearing Research, International Journal of Audiology, Acta Oto-Laryngologica*, and *European Archives of Oto-Rhino-Laryngology*. These journals have had a significant influence on otolaryngologists, audiologists, and neurologists across the world, and have impacted the orientation of research in their corresponding scientific fields. This trend indicates that tinnitus is one of the central issues in otolaryngology and neurology. The top 10 co-cited references for the period 2001–2020 indicated that researchers are more concerned with the clinical management of tinnitus. Notably, the first reference with the highest co-citation rate and landmark was the article “*Adding Insult to Injury: Cochlear Nerve Degeneration after ‘Temporary' Noise-Induced Hearing Loss”* published by Kujawa and Liberman (19), which proposed noise can cause progressive injury to the ear, and this neurodegeneration is supposed to increase hearing difficulties in noisy circumstances and may cause tinnitus.

Keyword co-occurrence analysis provides insight into the distribution and evolution of various research hotspots within a certain field. The top 10 high-frequency keywords in the co-occurrence cluster analysis showed that epidemiology, pathophysiological mechanisms, and treatments continue to be hot subjects in tinnitus research. Burst keywords indicate up-and-coming trends and research frontiers. Three leading areas of tinnitus research were identified: prevalence (2018–2020), anxiety (2017–2020), and network (2017–2020). Knowledge of the prevalence of a disorder in a specific population is important for improving health and preventing the disease. According to the US epidemiological survey report, 10–15% of patients worldwide are suffering from tinnitus (6). Several studies have reported a 16.9% prevalence of tinnitus in people aged 40–69 years in the UK (3). Findings from research in Egypt, Japan, and Nigeria suggest that tinnitus prevalence in these countries is approximately similar to that in Europe and the US (2). South Korean scholars found that the prevalence of tinnitus in South Korea was 19.7% (25). Tinnitus is normally accompanied by negative psychological responses such as sleep disturbances, irritability, inability to concentrate, anxiety, and depression. The emotional response induced by the initial phase of tinnitus can be regarded as a status of stress in response to unusual sounds. It is well-known that this stress state increases the sensitivity of the hair cells through sympathetic excitation, thus raising compensatory efficiency, which also generates a certain degree of anxiety and tension. This unpleasant emotional perception of tinnitus is delivered to the cerebral cortex, exacerbating the misunderstanding of tinnitus and leading to a vicious circle between tinnitus and negative emotions (26). At present, the mechanism of tinnitus perception is still uncertain. Numerous studies have shown that dysregulation of the forebrain and the limbic system is a neuro-related factor in tinnitus (27). More specifically, disruption of the anterior striatal network has been thought to contribute to tinnitus by impairing “noise cancellation” (28). The role and mechanism of neural networks in tinnitus remain to be further explored.

The treatment of tinnitus is always a point of interest for research by otolaryngologists, audiologists, and neurologists. Currently, the pathogenesis of tinnitus has not been fully elucidated, and its treatment lacks uniform standards. Several therapies are available to treat tinnitus, including behavioral therapies, tinnitus retraining treatment (TRT), surgery, and medications. Neuroimaging investigations have identified a number of neural systems related to tinnitus annoyance, which include noticing (dorsal and ventral), default mode, limbic, auditory, somatosensory, and visual brain networks (29). Tinnitus is heterogeneous in its biological foundation. Tinnitus annoyance probably associates with abnormal function of diverse networks in individual patients. These unusual networks probably result from continuous chronic abnormalities in auditory function in some patients, while others have pre-existing vulnerability. Based on the neural network theory, individual variations in the pathogenesis of tinnitus demand a precision medicine approach to treating individual patients (30). The first step toward precise healthcare will involve predicting which patients will react to a specific tinnitus therapy. The second step emphasizes pairing patients with a particular set of potential treatments.

From the timeline of the clustering diagram, we observed a novel focus on tinnitus in the form of hidden hearing loss (HHL), which is a pathology of the afferent pathway of the cochlea induced by noise exposure, drug damage, and/or aging. It has no impact on absolute hearing and has regular pure-tone hearing thresholds at the common frequencies, but exhibits only diminished speech recognition and decreased spatial localization in complicated environments (31). Patients suffering from hearing loss and tinnitus are able to receive benefits from hearing aids depending on the accurate assessment of hearing loss and tinnitus features. Nevertheless, tinnitus is not always accompanied by hearing loss. In daily work, pure-tone hearing thresholds are observed in octave or half-octave intervals ranging from 250 to 8000 Hz. This technique may risk missing the hearing loss over a narrow frequency bandwidth, known as HHL (22). Lefeuvre et al. (32) assessed hearing loss in 66 tinnitus patients with frequency accuracy and intensity accuracy at 1 dB HL using definition audiograms ranging from 1/24 octave to 1 Hz. The outcomes revealed that HHL was detected in 81.8% of the tinnitus patients; HHL was not detected in the volunteers without tinnitus. High-definition audiograms were utilized to detect HHL correlated with tinnitus and to establish the pitch and frequency of tinnitus. This allowed audiologists to capture precise information about hearing loss and tinnitus traits to enhance tinnitus management with hearing aids.

### Limitations

This bibliometric analysis provides a superior understanding of constantly changing hotspots and trends in research compared with conventional reviews, which are incapable of administering vast amounts of heterogeneous literature; nevertheless, it has some limitations. First, the WoSCC database is regarded as the most important source of data for bibliometric analysis (33), so we did not search other databases and some research may have been ignored. In some cases, the identified researchers might not have published original discoveries but instead published large numbers of reviews, which were then cited frequently. Some of the most cited references are not tinnitus specific, they are about methods applied to many problems including tinnitus, so have a wider audience, and do not represent current trends or hot spots. Besides, variation in the continuously updated database might lead to discrepancies between the search results and the actual number of included publications. In addition, the omission of books/chapters/letter and the consideration of articles published in English only may lead to some bias in the analysis. The last limitation is that this study cannot fully identify the role of authors in affiliations, and it is possible that they are honorary or part-time. Some of the authors may work at a university, but also have associations with the university where he used to work. Therefore, a more accurate literature analysis should be based on knowledge maps with bibliometric software, incorporating specific associated literature. However, we still believe that this work can be applied to present the overall situation and general trend in this field.

## Conclusion

Over the past two decades, the quantity of publications in the field of tinnitus has been growing at a rapid pace. At present, the recognition of tinnitus has improved significantly in all aspects. Articles published in journals dedicated to otolaryngology and neurology will attract more attention than those published in general journals. The US is the leader in this domain, having contributed almost 30% of all tinnitus papers worldwide, which has significantly facilitated the advancement of research. Therefore, collaboration between countries, institutions, and scholars should be strengthened. Tinnitus is a prospective field of study, and intensive research on tinnitus will benefit a wide spectrum of patients. Epidemiology, pathophysiology, treatment, and neural networks are the current research hotspots in the area of tinnitus.

## Data Availability Statement

The raw data supporting the conclusions of this article will be made available by the authors, without undue reservation.

## Author Contributions

FZ and GY designed the study. TZ interpreted the data. FZ wrote the article. YJ, YM, and ZX collected and analyzed the data. MZ checked the data. GY and TZ revised the article. All authors read and approved the final manuscript.

## Funding

This study was supported by the Science and Technology Foundation of Guizhou Province (D2011160) and the National Natural Science Foundation Cultivation Project of the Affiliated Hospital of Guizhou Medical University (gyfynsfc [2020]-7).

## Conflict of Interest

The authors declare that the research was conducted in the absence of any commercial or financial relationships that could be construed as a potential conflict of interest.

## Publisher's Note

All claims expressed in this article are solely those of the authors and do not necessarily represent those of their affiliated organizations, or those of the publisher, the editors and the reviewers. Any product that may be evaluated in this article, or claim that may be made by its manufacturer, is not guaranteed or endorsed by the publisher.
